# Antifungal activity of Taurolidine against Mucorales: An in vitro study on clinical isolates

**DOI:** 10.18502/cmm.8.1.9211

**Published:** 2022-03

**Authors:** Hadis Jafarian, Ali Amanati, Parisa Badiee

**Affiliations:** 1 Clinical Microbiology Research Center, Shiraz University of Medical Sciences, Shiraz, Iran; 2 Alborzi Clinical Microbiology Research Center, Shiraz University of Medical Sciences, Shiraz, Iran; 3 Department of Pediatrics, Division of Pediatric Infectious Diseases, School of Medicine, Shiraz University of Medical Sciences, Shiraz, Iran

**Keywords:** Minimum inhibitory concentrations, Mucormycosis, *Mucorales*, Taurolidine

## Abstract

**Background and Purpose:**

Taurolidine is active against a wide variety of micro-organisms, including bacteria and fungi. Mucormycosis is one of the life-threatening opportunistic fungal infections, especially in immunocompromised patients. Currently, the emergence of Mucormycosis during the COVID-19 pandemic raises public health concerns regarding untoward morbidity and mortality among SARS-CoV-2 patients. It is well-known that delayed and inappropriate antifungal therapy leads to increased morbidity and mortality. This study aimed to investigate the *in-vitro* antifungal activity of taurolidine to evaluate its effects against clinical isolates of *Mucorales*.

**Materials and Methods:**

This study included previously collected clinical *Mucorales* isolates. The minimum *in vitro* inhibitory concentration (MIC) of amphotericin B, caspofungin, voriconazole, posaconazole, and itraconazole was determined using the broth microdilution method.

**Results:**

All clinical isolates showed full sensitivity to amphotericin B. Posaconazole MIC range from 8 μg/mL to 0.032‎ μg/mL. The MIC range of voriconazole and caspofungin were determined to be 2-8 µg/mL and 0.5-16 µg/mL, respectively. Growth of the isolates was entirely inhibited in 1000 µg/mL concentration of taurolidine. In microscopic observations, morphological effects on hyphal growth were observed at 500 µg/mL concentration.

**Conclusion:**

In conclusion, this is an updated experience of using taurolidine against *Mucorales*. However, our in-vitro findings need to be confirmed in well-designed clinical trials aimed at treating invasive Mucormycosis infections.

## Introduction

Taurolidine (4-[(1,1-dioxo-1,2,4-thiadiazinan-4-yl) methyl]-1,2,4-thiadiazinan 1,1-dioxide) is derived from the amino acid taurine which is made naturally within the body. Taurolidine has antifungal, antibacterial, anticoagulant, and potential antiangiogenic activities [ 1 , 2 ]. Taurultam, taurinamide, and taurine are the main metabolites of taurolidine. Taurolidine molecule generates three methylol-containing fragments (methylol- taurultam, methylol-taurinamide, and taurultam), which are considered to be active derivatives with antibiotic and endotoxin properties through irreversible binding to the cell walls of organisms [ 3 ]. Methylol-containing moieties appeared to react with bacterial or fungal cell wall components to prevent the adherence of micro-organisms to biological surfaces, such as epithelial cells [ 1 , 4 ]. Taurolidine has antimicrobial activity against various bacteria and fungi and effectively prevents biofilm formation in central venous catheters [ 5 - 7 ]. To date, no antimicrobial resistance has been observed *in vitro* [ 8 ].

Mucormycosis is a life-threatening opportunistic fungal infection in immunocompromised hosts and certain metabolic diseases such as diabetes [ 9 ]. During the COVID-19 pandemic, rhino-orbital mucormycosis reemerged as an opportunistic infection [ 10 - 12 ].

Rhino-orbital, pulmonary, and cutaneous infections are the most common forms of mucormycosis [ 13 - 15 ]. Delayed and inappropriate antifungal therapy is one of the leading causes of morbidity and mortality (ranging from 41% to 52%) in the affected individuals [ 15 - 17 ]. Delayed antifungal therapy (>6 days) is associated with a significant increase (2-fold and even more) in the mortality rate of mucormycosis [ 18 ].

There are promising reports on taurolidine treatment of bacterial/fungal catheter-related bloodstream infections, including gram-positive, gram-negative, and *Candida* infections [ 5 - 7 ]; however, there is a lack of data on the anti-*Mucorales* activity of taurolidine. 

This study aimed to investigate the *in vitro* antifungal activity of taurolidine to evaluate its effects as an antifungal agent against clinical isolates of *Mucorales*.

## Materials and Methods

### Clinical isolates

A total of ten previously collected clinical *Mucorales* isolates were included in this study. The isolates of clinical samples obtained from Namazi and Amir hospitals, Shiraz, Iran, were referred to the mycology department at the Professor Alborzi Clinical Microbiology Research Center, Shiraz University of Medical Sciences, Shiraz, Iran. Samples were collected from different sites, including sinus and skin, by deep tissue biopsy from clinically symptomatic immunocompromised patients. A total of seven isolates were identified through amplification of the D1/D2 region and subsequent sequencing. Data were compared to the NCBI nucleotide database and deposited in GeneBank (accession number: MZ695808-11, MZ695830, MZ695831, MZ695844).

### Antifungal susceptibility testing

Susceptibility testing was performed using the CLSI M38-A2 [ 19 ]. All isolates were cultured on Sabouraud dextrose agar ‎ before susceptibility testing to ensure viability. Stock spore suspensions were prepared by washing the slant’s surface with 2 mL of sterile saline containing 0.05% Tween 80. Antifungal drugs were obtained from their respective manufacturers as standard powders. Stock solutions of amphotericin B (Sigma-Aldrich, Germany), itraconazole (Sigma-Aldrich, USA), posaconazole (Sigma-Aldrich, Germany), and voriconazole (Sigma-Aldrich, USA) were dissolved in dimethyl sulfoxide (Merck, Germany), and caspofungin (Sigma-Aldrich, USA) was dissolved in sterile water. The drug concentration ranged from 0.03 to 16 µg/mL in all compounds. Serial two-fold dilutions of the various drugs were prepared in Roswell Park Memorial Institute (RPMI) 1640 medium (with L-glutamine, without bicarbonate) (Sigma-Aldrich, USA) and buffered to pH 7.0 using a 0.165 M solution of MOPS (Sigma-Aldrich, USA). Spore suspensions were diluted into RPMI to a concentration of 2×10^4^ CFU/mL. MICs were determined in 96-well plates with conidial suspensions in RPMI. Inoculated plates were incubated at 35°C and read visually after 24 and 48 h. For amphotericin B, MIC was checked and recorded at 24 and 48 h to determine the concentration of the drug that elicited complete (100%) growth inhibition. Afterward, MICs were checked and recorded at 24 and 48 h to determine the growth inhibition (50%) for itraconazole, posaconazole, and voriconazole. Minimum effective concentration (MEC; the lowest concentration at which the morphological changes of fungal hyphae could be observed) was used to assess *in vitro* antifungal susceptibility of caspofungin to *Mucorales*. The ATCC 22019 strain of *Candida parapsilosis* was included as a control strain. To determine the *Mucorales* growth inhibition by taurolidine, NutriLock solution (TauroPharm, Germany) was used, which did not contain citrate or heparin. A serial dilution of taurolidine ranging from 2000 µg/ml to 3.9 µg/ml was prepared in RPMI. The clinical isolates were first cultured on Sabouraud dextrose agar at 35 ºC. Standard conidial suspensions were prepared at final concentrations of 2×10^4^ CFU/mL in RPMI. Subsequently, the conidial suspensions were added to serial dilutions of taurolidine which was previously prepared in RPMI and incubated at 35ºC. The inhibitory effect compared with the growth control well was evaluated visually and microscopically after 24 and 48 h. The MIC is defined as the lowest drug concentration at which complete inhibition of growth occurs.

## Results

Antifungal MICs for the quality control isolate were within the expected range. The *in vitro* activity of the five antifungal agents tested against the seven strains is summarized in Table 1. There was a slight difference between MIC data collected after 24- and 48-h incubation. Overall, amphotericin B and posaconazole were the most active drugs against tested organisms. For all the strains, the MIC of voriconazole and caspofungin were estimated at ≥2 µg/ml and 16 µg/ml, respectively.

**Table 1:** MIC and MEC (μg/mL) at 24 h and 48 h exposure of selected antifungals against tested strains

No.		AMP (µg/mL)	CAS (µg/mL)	VOR (µg/mL)	POS (µg/mL)	ITR (µg/mL)
1	*Rhizopus oryzae*	0.032	16	2	0.032	0.064
2	*Rhizopus oryzae*	0.032	16	2	0.032	0.125
3	*Rhizopus oryzae*	0.032	16	4	0.064	0.125
4	*Rhizopus oryzae*	0.032	16	4	0.064	0.125
5	*Saksenia vasiformis*	0.032	16	2	0.032	0.064
6	*Rhizopus microsporus*	0.032	16	2	0.032	0.125
7	*Rhizopus* spp.	0.032	16	4	0.064	0.125
8	*Rhizopus microsporus*	0.25	0.5	8	8	8
9	*Rhizopus* spp.	0.064	8	8	8	4
10	*Rhizopus* spp.	0.5	8	4	2	8

AMP: amphotericin B; CAS: caspofungin; VOR: voriconazole; POS: posaconazole; ITR: itraconazole.

^a^ Minimum Effective Concentration (MEC) is reported

**Figure 1:**
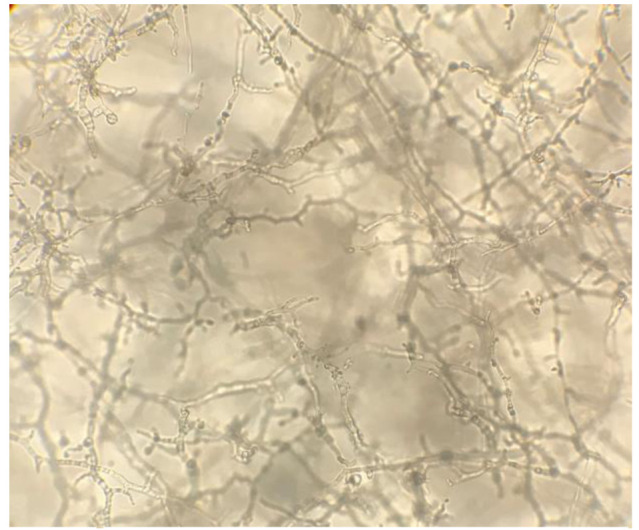
Light microscopic observations of tested isolates exposed to taurolidine. Dense and normal hyphal growth in control well

**Figure 2:**
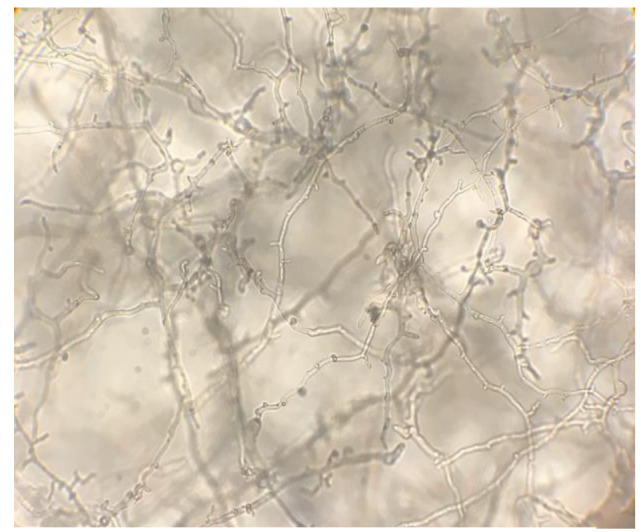
Slight morphological effect in 250 ‎µg/ml‎ concentration

**Figure 3:**
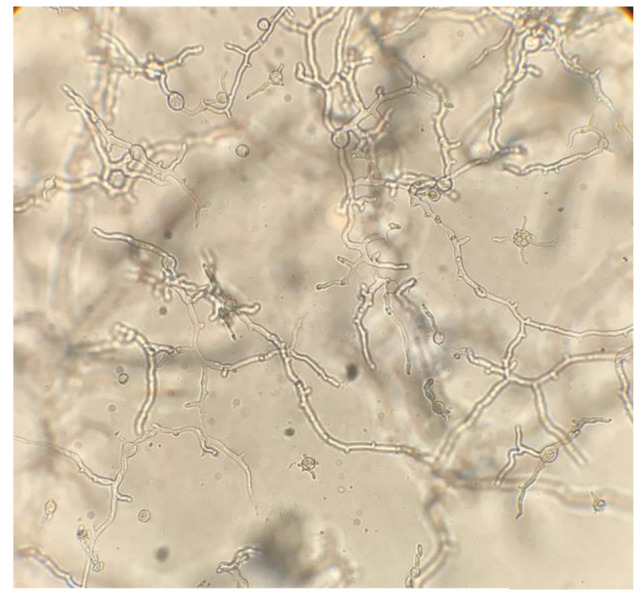
Short, distended, and balloon-shaped hyphae in 500 ‎µg/ml‎ concentration

**Figure 4:**
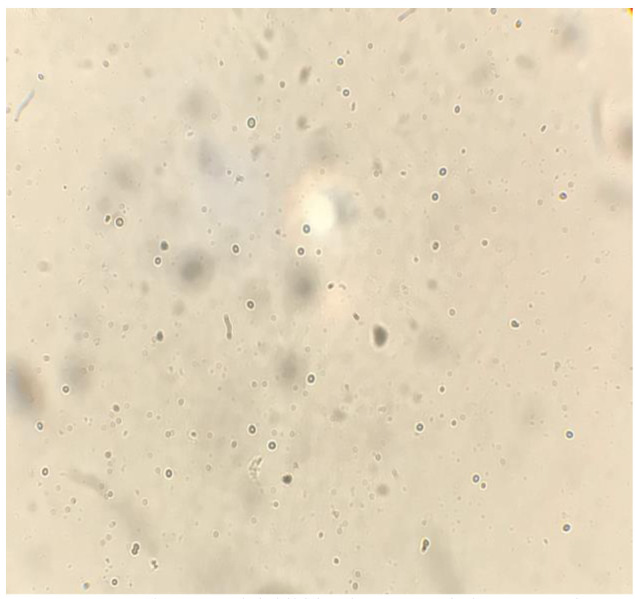
Complete growth inhibition in 1000 ‎µg/ml‎ concentration

On visual observation, the growth of isolates was completely (100%) inhibited at a 1000 µg/mL concentration of taurolidine. On microscopic observations, morphological effects on hyphal growth were observed at 500 µg/mL concentration compared to the controls (Figure 1-4). There was not any difference between reading time end-points at 24h or 48h.

## Discussion

Antifungal resistance developed rapidly due to the misuse and overuse of these agents [ 20 ]. Amphotericin B, posaconazole, and isavuconazole are available choices for treating mucormycosis [ 21 , 22 ]. Liposomal formulation of amphotericin B is the first-line recommended antifungal, while intravenous isavu-conazole and intravenous or delayed-release tablet posaconazole are considered alternative choices, especially in those with preexisting renal diseases [ 22 ]. The antifungal MIC distributions of azoles and amphotericin B reported here for members of the *Mucorales* are similar to those in previous studies [ 16 , 23 ]. Based on obtained results, taurolidine efficiently inhibited the growth of *Mucorales* *in vitro* in this study comparable with other antifungals active against *Mucorales*. Taurolidine, a taurine derivative, is a known antibacterial adjuvant that is successfully used during surgery in cases of peritonitis to reduce the severity of inflammatory peritoneal adhesions [ 24 ], for lavage of the wounds, and difficult-to-treat cases of osteomyelitis [ 25 ]. 

In their study, Bosch et al. estimated a 0.30 pooled incidence rate ratio with a confidence interval of 0.19-0.46 for 918 patients in nine studies that favored taurolidine-containing lock solutions. They also reported mild and scarce adverse events [ 26 ]. In another study, Roden et al. reported that removal of central venous catheters due to infection or catheter malfunction occurred less often in the presence of taurolidine-based lock solutions [ 27 ].

In addition to the chemical reaction with bacterial cell walls to prevent adhesion of the bacteria to the biological surfaces, taurolidine was documented to have anti-inflammatory activities. 

Ezzat et al. reported that the use of taurolidine for temporary hemodialysis catheters was associated with lower inflammation markers, lower incidence of catheter-related bloodstream infections, and better catheter performance [ 28 ].

Taurolidine is shown to have anti-interleukin-1 and anti-tumor necrosis factor-alpha activity in *in vitro* and *in vivo* studies [ 29 , 30 ]. In addition, it is effective against catheter-related bloodstream infections, even in the presence of biofilms [ 31 ]. Several reports confirmed that taurolidine could successfully prevent central venous catheter microbial colonization and infections, including a wide range of gram-positive and gram-negative pathogens, and some fungi, such as *Candida* spp. [ 5 , 7 , 32 , 33 ]. 

Taurolidine is a safe antimicrobial agent; however, reversible thrombocytopenia and neutropenia are associated with intravenous use [ 34 ]. Moreover, localized pain was when taurolidine was administered to pediatric cancer patients via a peripheral cannula [ 35 ]. No other significant side effects have been reported from either intravenous or intraperitoneal use [ 36 ]. Taurolidine is compatible with other medications when used concurrently [ 37 ]. 

In the present study, caspofungin demonstrated complete resistance against tested organisms except for one organism, which also demonstrated lower MIC against taurolidine. There are reports of using combination therapy with amphotericin B and either posaconazole or an echinocandin for the treatment of *Mucormycosis* [ 15 , 16 , 38 ]. 

Based on our microscopic observation, the taurolidine mechanism of action may in some ways be similar to echinocandins. Taurolidine can bind to cell wall polysaccharides [ 2 , 39 ]. In our study, short, distended, and balloon-shaped hyphae were observed in 500 ‎µg/mL‎ concentration of taurolidine, which may indicate cell wall disruption. Gorman found that bacterial cells grown in the presence of sub-inhibitory concentrations of taurolidine lost their ability to complete cell division and appeared filamentous [ 4 ]. 

In previous studies, taurolidine solution resulted in the significant killing of *Candida albicans* [ 34 ]. Shah reported the fungicidal activity of taurolidine-citrate solution against *Candida albicans* at 135 µg/mL concentration after 24 h exposure [ 40 ]. Olthof and his coworkers also found that various taurolidine-containing dilutions could prevent the growth of *Candida glabrata* *in vitro* [ 41 ]. In our study, fungicidal activity of taurolidine was observed at 1000 µg/mL concentration, and morphological effects were observed at 500 µg/mL concentration. The effect of taurolidine on fungal structures seems to be concentration-dependent. It was able to eradicate all *Mucorales* in 1000 µg/mL concentration. 

Accordingly, taurolidine could be considered a highly effective wound-compatible antifungal and antibacterial agent. While the consensus on wound antisepsis (last updated in 2018) does not currently recommend taurolidine as effective wound antisepsis, our findings provide new insight regarding taurolidine activity against *Mucorales*, which could be helpful in the local treatment of difficult-to-treat invasive mucormycosis infection of the skin and soft tissue [ 42 ]. 

Moreover, in another study, topical taurolidine was used against rabbit experimental *Staphylococcus aureus* keratitis. The results suggested that topical taurolidine was an effective ocular chemotherapeutic agent [ 43 ].

Regarding the limitations of this study, one can refer to the lack of access to an electron microscope for a better understanding of structural changes. Probable differences in sensitivity to taurolidine between species can also be evaluated in the future. Moreover, we analyzed relatively few clinical strains; therefore, generalization of the results should be done with caution. However, our findings might help researchers in future studies.

## Conclusion

This study was an updated experience of using taurolidine against *Mucorales* which confirmed the antifungal activity of taurolidine. Our results may have generated valuable data regarding the alternative antifungal agents for the treatment of invasive mucormycosis. However, further clinical studies should be conducted to investigate their potential clinical efficacy against *Mucorales* infections.

## Acknowledgments

Our thanks go to Professor Alborzi Clinical Microbiology Research Center staff for their technical support and assistance.

## Authors’ contribution

AA was responsible for the organization and coordination of the trial. AA, HJ, and PB were the chief investigators and were responsible for the data analysis. AA, HJ, and PB conducted the study design. All authors contributed to the writing of the final manuscript and managed/ administered the trial. 

## Conflicts of interest

The authors declare that they have no known competing financial interests or personal relationships that could have influenced the work reported in any way.

## Financial disclosure

The authors received no financial stableupport/ funding for this study.
